# Effects of Salinization, Oil Contamination, and Heavy Metals on Soil Biological Activity and Phytoremediants

**DOI:** 10.3390/toxics14020186

**Published:** 2026-02-23

**Authors:** Gulnas Rafikova, Svetlana Mukhamatdyarova, Elena Kuzina, Liliya Kulbaeva, Milyausha Iskuzhina, Tatyana Korshunova

**Affiliations:** Ufa Institute of Biology, Ufa Federal Research Center, Russian Academy of Sciences, Ufa 450054, Russia; rgf07@mail.ru (G.R.); svetrm@gmail.com (S.M.); misshalen@mail.ru (Е.К.); l.kulbaeva78@mail.ru (L.K.); ishmurzina82@mail.ru (M.I.)

**Keywords:** carbonates, sulfates, copper, nickel, lupine, oats

## Abstract

Using plants to restore soils subjected to salinization and polychemic pollution can be an effective way to return agricultural land to circulation and obtain safe products. In this study, experiments were conducted with oats and lupine to evaluate their ability to purify soils contaminated with copper (II) and nickel (II) ions, carbonate and sulfate anions and oil and their combinations. The biological activity of the soil, phytotoxicity, and hydrocarbon content, as well as plant growth and biochemical parameters in polluted soil, were studied. The enzymes most sensitive to soil contamination were catalase, urease, and phosphatase. Copper ions inhibited oat root growth by 45.7% and lupine by 46.6%. Oil and its mixtures with other pollutants inhibited shoot growth by up to 50.3% in oats and up to 28.6% in lupine. The content of malonic dialdehyde increased in oats when exposed to copper, while in lupines, it increased 2.9-fold when exposed to oil. Flavonoids in oats increased with metal contamination (by 9–16.7%), while in lupines with oil (by 8.6%). Chlorophyll fluctuations were less pronounced in oats than in lupine. Despite the stress experienced by plants due to soil pollution, the degradation rate of petroleum hydrocarbons under oat and lupine crops was 33–46%. In general, oats and lupine are promising for the phytoremediation of complexly polluted and saline soils.

## 1. Introduction

High demand for oil on the global market has led to continued high rates of hydrocarbon production. Consequently, environmental pollution from oil and related pollutants remains a pressing issue and a source of serious concern.

In soil, hydrocarbons alter its physicochemical properties [1] and biological activity [2], and also have a negative impact on plant growth and development [3]. The presence of formation water in crude oil leads to soil salinization. Excessive concentrations of salt ions reduce the activity of soil enzymes [4,5], the intensity of nitrogen mineralization [6,7], the production of microbial biomass [8], and the rate of decomposition of organic matter [9].

Oil spills can release heavy metals into ecosystems. Heavy metals accumulated as a result of the excessive use of mineral fertilizers and pesticides can be found in arable soils contaminated with oil [10]. Many heavy metals are potentially toxic to living organisms at elevated concentrations [11]. Unlike organic matter, they are not subject to biological decomposition, but can only change their oxidation state and have a half-life of over 20 years [12]. Excessive accumulation of heavy metals in soils can have a negative impact on soil microorganisms [13], plants [14], and their functional activity [15].

Various remediation methods are used to clean up oil-contaminated soils. Phytoremediation is one of the most optimal and popular methods. However, predicting its effectiveness in the presence of additional pollutants is quite difficult. The interaction of several pollutants can contribute to changes in the physicochemical conditions of the soil due to the formation of various soluble and insoluble complexes, which, in turn, can affect the growth and metabolism of plants and rhizosphere microorganisms [8]. In this regard, studying the effect of combined pollution on the biological parameters of the soil, as well as on the state of remediated plants, is highly relevant. Previously, studies were conducted on the phytoremediation of oil-contaminated soils in the presence of salts or heavy metals [16,17]. However, there are very few studies devoted to a comprehensive study of the biological activity of the soil and the physiological state of phytoremediators during soil contamination with oil and associated pollutants [18,19].

The goal of the work was to study the impact of oil, copper ions, nickel, carbonate and sulfate salinization, and complex pollution (oil in combination with each of the listed pollutants) on the biological activity of podzolized chernozem, as well as on the morphometric and biochemical indicators of oats and lupine. It was suggested that the data from this study would allow us to assess the nature of changes in soil biological activity and the degree of abiotic stress experienced by phytoremediants in the simultaneous presence of pollutants of different chemical natures (oil and heavy metals, oil and salts). Expanding knowledge about the impact of complex pollution’s influence on various aspects of plant physiology and soil condition will provide new information necessary for the development of effective phytoremediation methods for the restoration of anthropogenically disturbed territories.

## 2. Materials and Methods

### 2.1. The Number of Soil Microorganisms

The serial dilution method was used to quantify the number of microorganisms in the soil. The number of microorganisms was determined by plating on the following mediums: heterotrophic microorganisms—nutrient agar [20], hydrocarbon-oxidizing microorganisms—Raymond agar [21] (0.1 g of sterile diesel fuel was applied to its surface), and micromycetes—Czapek’s medium [20]. All reagents used in this study were manufactured in Russia (JSC "Chemical Plant named after L.Y. Karpov" (Mendeleevsk), JSC Ural Plant of Chemical Reagents (Verkhnyaya Pyshma), LLC Salavat Plant of Chemical Reagents (Salavat).

### 2.2. Soil Enzymatic Activity

The activity of soil enzymes was analyzed as follows: catalase was determined by the gasometrical method by measuring the volume of oxygen released during the decomposition of hydrogen peroxide; invertase was analyzed by the colorimetric method by determining the content of reducing sugars; urease was determined by the colorimetric method by forming colored complexes with Nessler’s reagent; and phosphatase was analyzed by the colorimetric method by determining the organic fraction of hydrolyzed sodium phenolphthalein phosphate [22].

### 2.3. Soil Basal Respiration

Soil basal respiration was estimated using the ISO 16072 (2002) method [23]: 20 g soil samples (60% moisture content) were placed in gas-tight 900 cm^3^ vessels and incubated for 24 h at 20 °C. CO_2_ was absorbed with 0.05 M NaOH and precipitated as BaCO_3_ with 0.5 M BaCl_2_ solution. Unused NaOH was titrated with 0.1 M HCl adding phenolphthalein indicator, and then the amount of CO_2_ was calculated [24].

### 2.4. Plant Growth Conditions

The laboratory experiment was conducted using a randomized scheme. Scheme parameters: (1) 9 types of contamination: Cu^2+^, Ni^2+^, CO_3_^2-^, SO_4_^2^, oil, oil and Cu^2+^, oil and Ni^2+^, oil and CO_3_^2−^, oil and SO_4_^2−^. They were added to vessels (0.5 L) with 400 g of a mixture of soil (9 parts) and sand (1 part). Oil was added at a rate of 20 g/kg. Carbonate and sulfate salinization were simulated by adding aqueous solutions of sodium carbonate and magnesium sulfate, respectively. Each salt was added at a rate of 0.25% of the soil mass. For soil contamination with heavy metal cations Cu^2+^ and Ni^2+^, aqueous solutions of copper sulfate and nickel sulfate were used, respectively. Cu^2+^ was added at a concentration of 660 mg/kg, Ni^2+^—at 400 mg/kg. The selection of pollutant doses was related to the tolerance of the selected phytoremediators to these concentrations. The same concentrations of oil and anion salts as previously used [25] were chosen for this experiment. The concentrations of heavy metal cations were selected based on the critical values for copper and nickel at which plants can perform their bioremediation functions of contaminated soils; (2) two plant species were used: oats (6 sprouted seeds/vessel) and white lupine (5 sprouted seeds/vessel). Spring oat (*Avena sativa* L) variety Rysak No. 9463846 and lupine (*Lupinus albus* L.) variety Dega No. 9610203 included in the database “State Register of Varieties and Hybrids of Agricultural Plants Approved for Use” (Russia, https://gossortrf.ru/registry; accessed on 15 December 2025). The seeds were provided by the Bashkir Research Institute of Agriculture of the Ufa Federal Research Center of the Russian Academy of Sciences (Russia, Ufa). These species are often used for phytoremediation [26,27,28,29]. Previously, it was studied that they are resistant to oil and herbicides [19]; (3) 10 tests were conducted for each plant and type of contamination: (1) control (soil without contaminants); (2) tests with each of the 9 types of surface contamination. The tests were carried out in triplicate. In those variants to which solutions of solids or heavy metals were not added, the required amount of distilled water was added.

Growing conditions: 21 days, temperature 22–24 °C, controlled light (14 h photoperiod: photon flux intensity of 240 μmol m^−2^ s^−1^ PAR), and humidity 60% of the total soil water capacity. On the 14th day of the experiment, the concentrations of chlorophyll, flavonoids, and malondialdehyde (MDA) in plant leaves, as well as the nitrogen balance index (NBI), were analyzed. After the experiment, soil enzymatic activity, basal soil respiration, soil hydrocarbon content, soil phytotoxicity, and plant morphometric parameters (shoot and root length) were assessed.

### 2.5. Contents of MDA

The leaves of the plants were ground in trichloroacetic acid (10%) and then centrifuged (10,000× *g* rpm). The MDA content in the extract was determined by measuring the optical density of its complex with thiobarbituric acid [30].

### 2.6. Pigment Content and NBI

To measure the content of chlorophyll (*a* + *b*), flavonoids, and the NBI, a DUALEX SCIENTIFIC+ device (FORCE-A, Centre Universitaire Paris-Sud, Paris, France) was used according to the device’s instructions.

### 2.7. Content of Total Petroleum Hydrocarbons

The total petroleum hydrocarbon (TPH) content in the soil sample was measured using the EPA 3540C method using hexane as the extractant. The extract was evaporated to a final volume. The concentrated solution was dried to constant weight. The TPH content in the sample was determined gravimetrically.

The degree of TPH biodegradation was determined using the following formula: [(initial TPH content − final TPH content/initial TPH content] × 100.

### 2.8. Phytotoxicity

The phytotoxicity of the soil aqueous extract was determined using test plants radish (*Raphanus sativus* L.) of the Pink-Red variety. The seeds were germinated at 22 °C for 72 h. The percentage of seed germination, root elongation, and germination index were calculated according to [31].

### 2.9. Statistical Analysis

Statistica software (v.10) was used to process the data. Data are presented as mean ± standard error. The significance of differences was assessed using analysis of variance (ANOVA) followed by Duncan’s test. Statistical significance was set at *p* < 0.05.

## 3. Results

### 3.1. The Number of Soil Microorganisms

#### 3.1.1. The Number of Microorganisms in Soil Under Oats

The number of heterotrophic microorganisms in the soil with heavy metals and salt anions was comparable to the control values (Table 1). The presence of copper and nickel in the soil decreased the number of hydrocarbon-oxidizing microorganisms by 10–20 times. Copper addition led to a 20-fold increase in the number of micromycetes. Oil in the soil, both as a single pollutant and in combination with other pollutants, increased the population density of all studied microorganism groups.

#### 3.1.2. The Number of Microorganisms in Soil Under Lupine

As in the case of oats on oil-contaminated soil, the amount of all studied groups of microorganisms increased in oil-contaminated soil with lupine. Combinations of oil and heavy metals promoted the most significant (10–15 times) growth of the hydrocarbon-oxidizing microorganism population. In contrast to the variants with oats, a slight decrease in the number of micromycetes (approximately 2.5 times) was observed under lupine crops.

### 3.2. Soil Enzyme Activity

**Figure 1:**
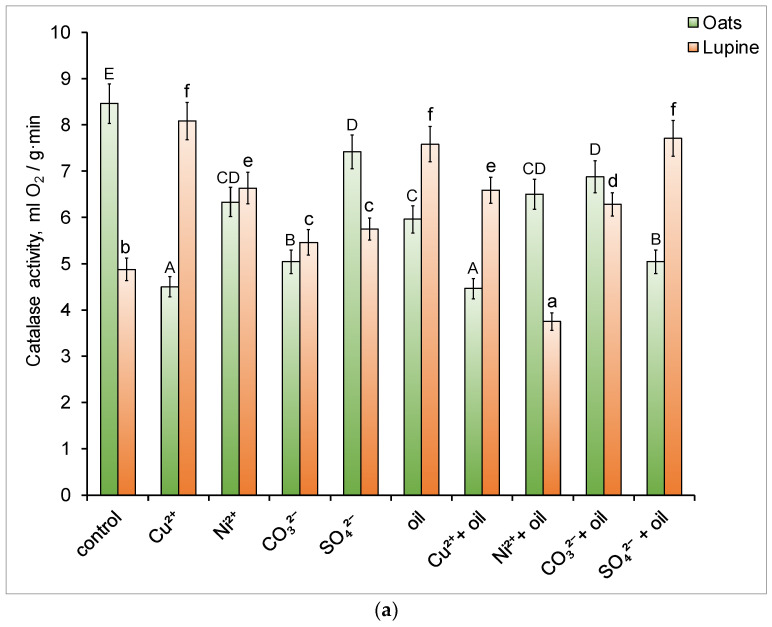

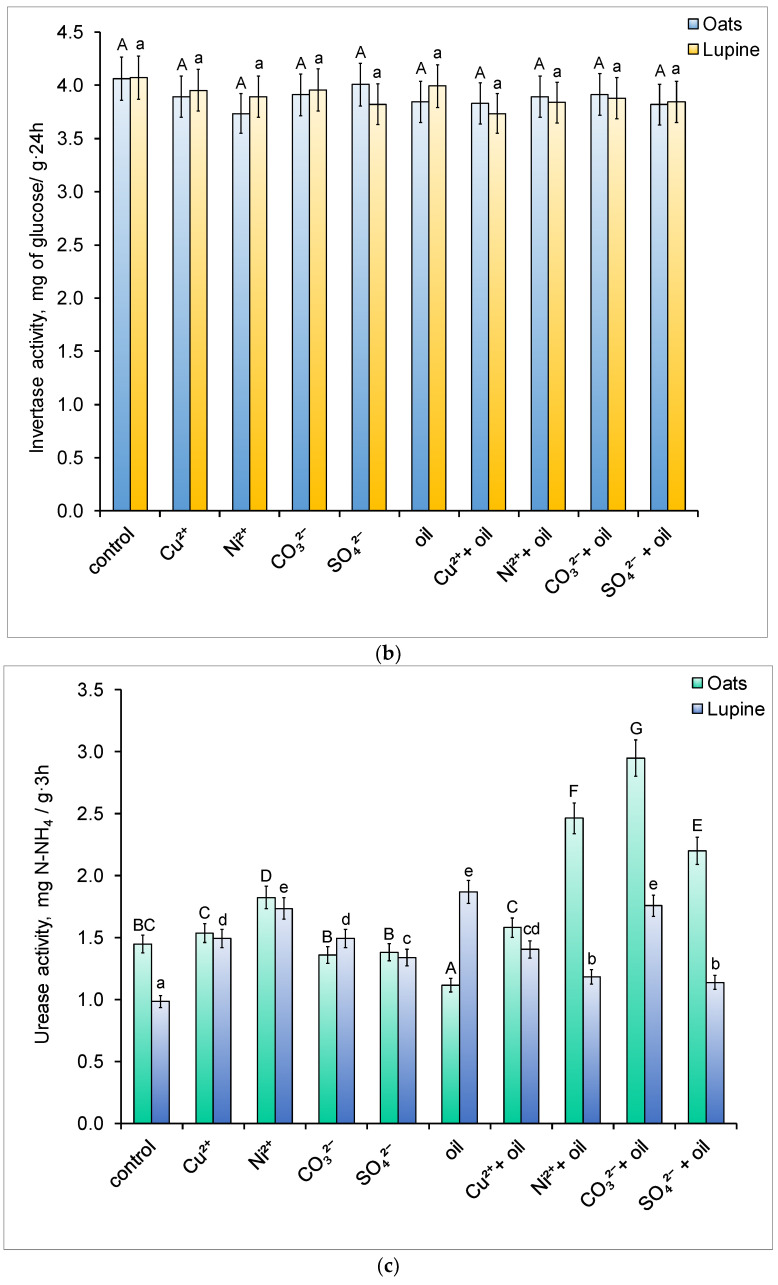

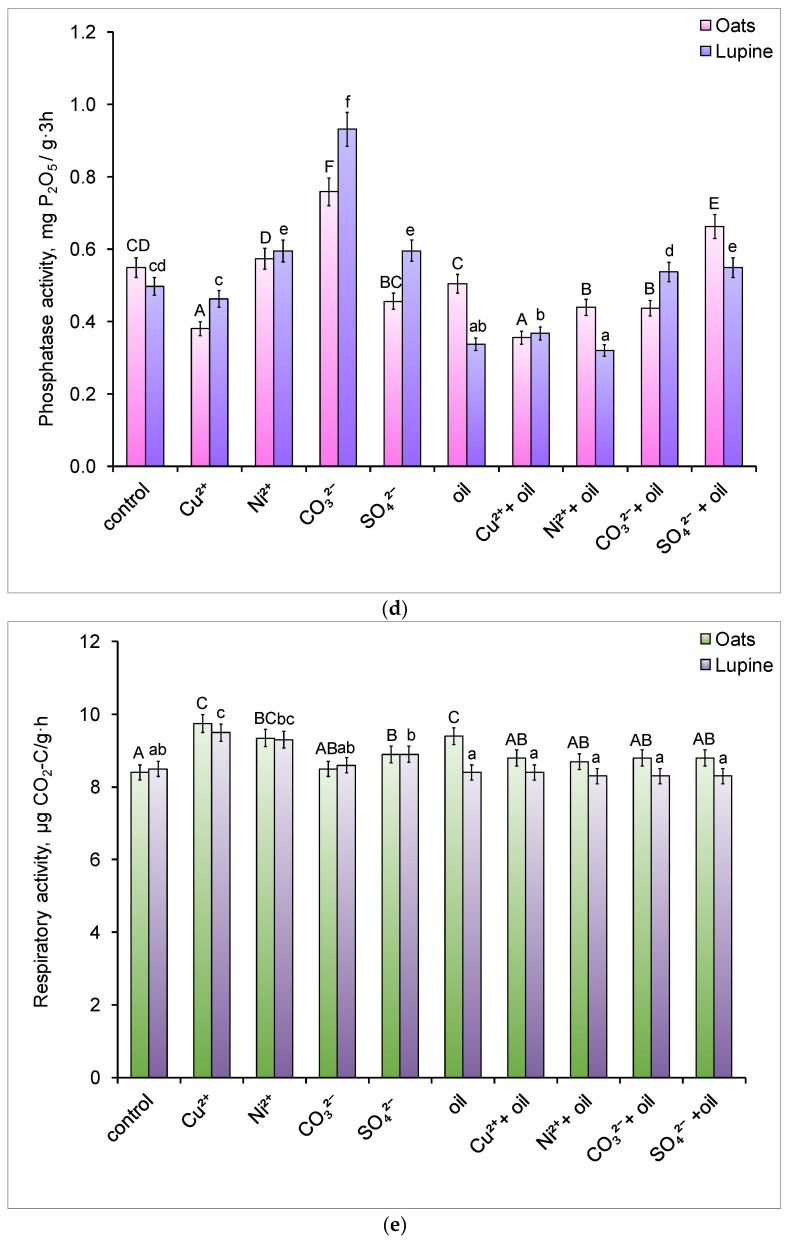
Enzymatic and respiratory activity in the soil under plant cultures: catalase activity (**a**), invertase activity (**b**), urease activity (**c**), phosphatase activity (**d**), and respiratory activity (**e**) after the experiment. Different letters indicate significant differences in the average values for each parameter (uppercase for oats, lowercase for lupine; *p* ≤ 0.05).

All types of pollutants under oats negatively affected soil catalase activity (CA), decreasing it by 12.3–47.3% (Figure 1a). Copper, both individually and in combination with oil, had the most suppressive effect. In contrast to the soil with oats, the CA value in the soil with lupine increased by 11.9–65.6% in the presence of pollutants. Only in the “oil + nickel” variant was CA lower than the control value.

Mono- or binary contamination did not suppress invertase activity (IA) in soil containing oats or lupine. IA in soil under plants ranged from 3.73 to 4.07 mg glucose/(g 24 h). No significant differences were observed between the values (Figure 1b).

The activity of urease (UA) in the soils under oats decreased by 22.8% after the addition of oil (Figure 1c). However, its combined use with nickel, carbonate ions, and sulfate ions led to a significant increase in UA. The activity of urease in the soils under lupine differed from that observed during oat stands (Figure 1c). All types of contaminants stimulated UA; in particular, the addition of oil led to an almost twofold increase in this parameter.

Phosphatase activity (PA) in the soil with oats was most strongly inhibited by copper ions (Figure 1d). When soil was contaminated with copper, PA decreased by 30.7%, and when copper and oil were combined, it decreased by 35.2%. Oil as a single contaminant did not significantly affect this parameter. However, its combined effect with heavy metals and carbonate anions inhibited PA. In soil with lupine, the maximum decrease in PA was observed with oil pollution (by 32.1%) and its combination with nickel (by 26.1%) or copper (35.7%) (Figure 1d). Heavy metals as monocontaminants did not exhibit an inhibitory effect on PA. Salinization, overall, had a positive effect on PA under lupine crops, including in the presence of oil in the soil.

### 3.3. Soil Basal Respiration

Soil basal respiration (SBR) under both plant species was similar—8.4–8.5 μg CO_2_-C/g·h (Figure 1e). Heavy metals caused the greatest increase in SBR (by 11–16% under oats crop and under lupine—by 9–12%). Oil induced an increase in respiratory activity only in soil under oat stands. Combined contamination did not have a significant effect on this indicator.

### 3.4. Morphometric Parameters of Plants

#### 3.4.1. Length of Shoots and Roots of Oat Plants

**Figure 2:**
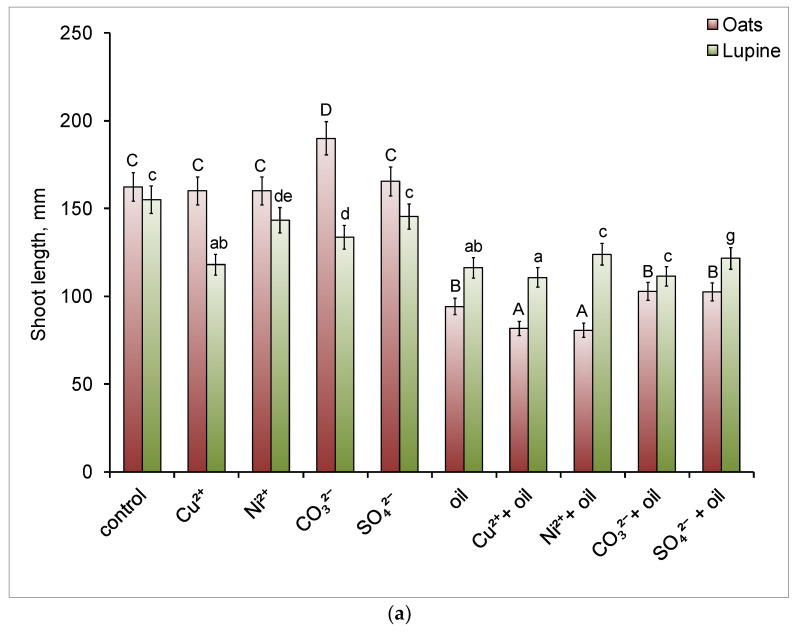

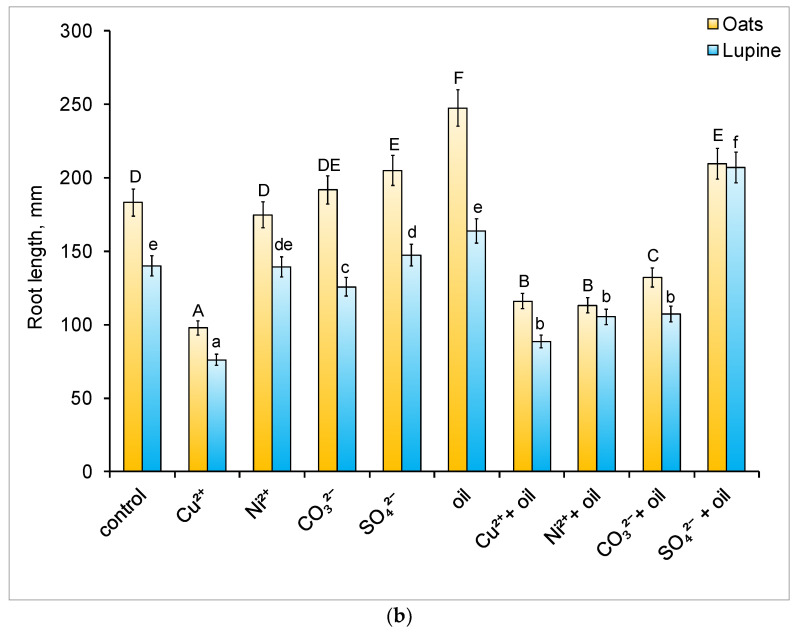
Shoot (**a**) and root (**b**) length of oat and lupine plants after the experiment. Different letters indicate significant differences in the average values for each parameter (uppercase for oats, lowercase for lupine; *p* ≤ 0.05, *n* = 15).

Soil contamination with copper and nickel, as well as the presence of sulfate ions, did not have a toxic effect on the formation of the aboveground part of oats (Figure 2a).

Under carbonate salinization, shoot elongation by 17% was observed. In the presence of oil, including in combination with heavy metals, their length decreased by 42.0–50.3%. With simultaneous contamination with oil and salts, shoot length was 60% lower than the control values.

Only copper had a negative effect on oat root length in soil without oil (Figure 2b); it decreased by 46.6%. Oil increased root growth by 35.1%. Binary contamination reduced root length more than only oil contamination, especially the combination of oil and heavy metals. Root length was shorter than that of the control plants by 27.9–33.9%, with the exception of the “oil + sulfate salinity” condition. In this case, root length was 14.3% greater than that of the control.

#### 3.4.2. Length of Shoots and Roots of Lupine Plants

Pollutants inhibited lupine shoot growth in all treatment variants except for the “nickel” and “sulfate ion” variants (Figure 2a). The greatest negative effect on shoots was caused by oil pollution (alone and in combination with other pollutants). Thus, with combined oil and copper pollution, this indicator decreased by 28.6%. Similar to oats, the presence of nickel in the soil did not negatively affect the morphometric parameters of lupine roots (Figure 2b) and shoots. Copper maximally inhibited root growth—their length was 45.7% less than the control, while oil and its combination with sulfate ion increased this parameter by 17 and 47.8%, respectively.

### 3.5. Malonic Dialdehyde Content

**Figure 3:**
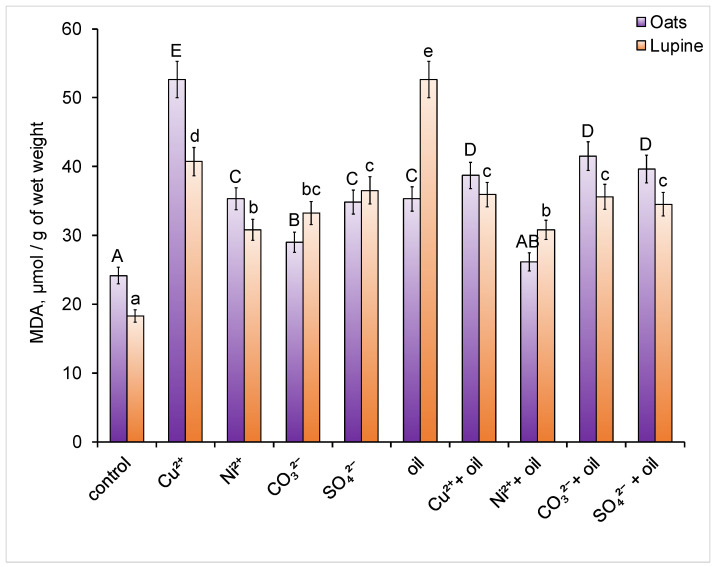
Malondialdehyde (MDA) content. Different letters indicate significant differences in the average values for each parameter (uppercase for oats, lowercase for lupine; *p* ≤ 0.05, *n* = 9).

With all types of pollution, the malondialdehyde (MDA) content in plant leaves in two species increased (Figure 3).

In contaminated soil, the maximum MDA content in oat leaves was found in the presence of copper (2.2 times higher than the control), while a significant minimum value was observed in plants grown in soil with carbonate. Lupine plants reacted to copper addition to the soil similarly to oat plants—the MDA content in the leaves increased by 2.2 times compared to the control. However, the largest increase in MDA (2.9 times) in lupine was observed with oil contamination. The minimum value of this indicator in contaminated soil was found in plants grown in the presence of nickel or its combination with oil.

### 3.6. Pigment Complex and Nitrogen Balance Index

The chlorophyll content in oat plants decreased slightly with soil contamination by nickel and oil, as well as in the “oil + nickel” and “oil + sulfate anion” treatments (by 7.2–8.6%) (Table 2). The maximum amount of this pigment was found in the leaves of plants in soil contaminated with carbonate and a combination of copper with oil (23.1 and 23.6 μg/cm^2^).

All types of oil soil contamination had a pronounced inhibitory effect on chlorophyll formation in lupine plants. A decrease in chlorophyll content in lupine plants was observed in the treatment with nickel added to the soil (by 11.38%) and in all types of oil contamination (by 18.42–41.27%). The greatest decrease in this parameter was observed in the treatment with oil addition.

The flavonoid content in oat leaves increased with the application of copper, both individually and in combination with oil (by 9–16.7%) (Table 2). Oil and salt anions, as single pollutants and in combination, reduced the contents of flavonoids in oat plants by 7.7–18%.

All types of pollutants in lupine plants suppressed flavonoid production. Only oil pollution increased flavonoid production by 8.6% compared to the control.

Oats in soils with carbonate or sulfate ions had the highest NBI values (Table 2); simultaneous salinization and oil contamination did not reduce it compared to the control. The largest decrease in this indicator (17.5%) was observed with copper contamination. Oil as a monopollutant had no effect on the NBI in oat plants. Conversely, in lupine, the NBI decreased by 17.9–39.3% in all oil treatments, except for combinations with nickel.

### 3.7. Total Hydrocarbon Content in Soil

**Figure 4:**
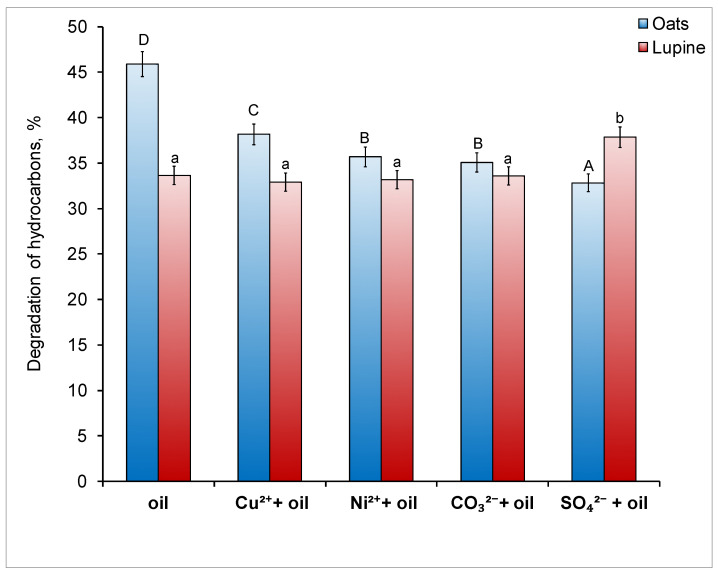
The degree of hydrocarbon degradation in the soil under oat and lupine crops after the experiment. Different letters indicate significant differences in the average values for each parameter (uppercase for oats, lowercase for lupine; *p* < 0.05).

Overall, hydrocarbon degradation in soils under oat and lupine stands was relatively high, reaching 33–46% and 34–38%, respectively (Figure 4). The presence of additional pollutants reduced the rate of oil degradation only in soils under oats by 7.7–13.1%, whereas no such effect was observed in soils under lupine. In contrast, sulfate addition stimulated oil degradation.

### 3.8. Phytotoxicity

Copper and its combinations with oil more noticeably suppressed the germination and root elongation of radish under the stands of both plants, compared to the variant’s “nickel” and “nickel + oil” (Table 3). Carbonate salinization, unlike sulfate salinization, did not exert such a significant inhibitory effect on these parameters in the soil under oats stands. Combinations of both salts with oil similarly affected the germination of the test plant.

Overall, the phytotoxicity of contaminated soils 21 days after the start of bioremediation was moderate or completely absent.

## 4. Discussion

Phytoremediation is an economically efficient and environmentally safe approach to soil remediation. It enables the removal or transformation of pollutants into less toxic or biologically unavailable forms through plant physiological processes, as well as microbial activity in the rhizosphere [32]. The use of local plant species is considered preferable, as they are adapted to the specific climatic and soil conditions of the region [33]. Most phytoremediation studies focus on soils contaminated with a single xenobiotic, whereas anthropogenic pollution is typically polychemical in nature [34]. This complicates remediation due to the synergistic effects of pollutants, which may enhance their negative impacts. In the present study, oat (*Avena sativa* L.) and white lupine (*Lupinus albus* L.) stands were used as phytoremediators—plant species widely distributed in temperate climates. These species have been employed as remediation agents for soils contaminated with oil and heavy metals [35,36]. Previous studies conducted by the authors demonstrated that oats and lupine exhibit tolerance to oil, herbicides of various classes, heavy metals, and excessive chloride concentrations [19,25]. The present study focused on investigating the effects of single contamination (with oil, copper, nickel, carbonate ions, and sulfate ions), combined contamination (oil in combination with each of the listed pollutants), and phytoremediation on soil biological activity, including microbial abundance, soil enzyme activities, and soil basal respiration. Furthermore, it was of interest to evaluate the effectiveness of the phytoremediation of oil-contaminated soil in the presence of additional pollutants, based on the degree of oil biodegradation. Another important aspect of this study was the analysis of the morphological and biochemical parameters of oats and lupine, including the root and shoot length, pigment content, and MDA levels, which enabled the assessment of plant responses to pollutant exposure, including their combined effects. Ultimately, the obtained results will allow us to assess the feasibility of using phytoremediants for the remediation of soils subjected to complex contamination and to gain new insights into its effects on higher plants.

Microorganisms are an essential component of soil ecosystems and play a key role in determining soil biological activity. Due to their high sensitivity to external factors, they can serve as indicators for assessing environmental quality [37].

In general, none of the studied types of soil contamination under oat and lupine stands exerted a pronounced toxic effect on the soil microbiome. The negative impact of pollutants on microorganisms may, on the one hand, have been mitigated by the high buffering capacity of chernozem soils [38] and, on the other hand, alleviated by the development of plant root systems. Plant roots release exudates that serve as a nutrient source, enhance soil aeration, and provide habitats for microbial colonization [39]. In addition, oil at the applied concentration could act as an additional carbon source for the studied microbial groups. Consequently, their abundance increased in the presence of oil and its combinations with other contaminants. In the case of combined contamination with oil and heavy metals, this effect may also be associated with a reduced proportion of fungi in the microbial community and, consequently, an increased role of bacteria.

Enzyme activity is an important indicator of soil condition. Enzymes produced by soil microorganisms are directly involved in the decomposition of cellulose and other plant residues, as well as in the transformation processes of nitrogen, phosphorus, and sulfur. Oxidoreductases (dehydrogenases, catalase) and hydrolases (phosphatase, urease, arylsulfatase, and β-glucosidase) play particularly significant roles. Changes in enzyme activity often precede biological transformations in soil and can serve as indicators of anthropogenic contamination.

Catalase activity is closely associated with the abundance of aerobic microorganisms and the soil’s capacity for self-purification under contamination conditions [40]. A significant decrease in CA under oat stands following soil contamination with copper ions (Figure 1a) can be attributed to the adverse effects of excessive concentrations of this heavy metal on root system development. This, in turn, negatively affected the survival conditions of soil microorganisms, thereby influencing their enzymatic activity. Furthermore, oats, when exposed to copper contamination in soil, release phenolic compounds [41], which have selective antimicrobial activity [42]. Phytosiderophores such as mugic acid are released by cereal roots [43], and may also act as antimicrobial agents. The potential suppression of certain rhizosphere microbial species could have led to a decrease in CA.

In contrast, an increase in CA under lupine stands may be associated with root exudation. Lupine roots are known to release substantial amounts of low-molecular-weight organic acids (primarily malates and citrates) into the soil. These compounds serve as carbon sources for rhizosphere microorganisms and may also function as signaling molecules involved in plant–microorganism interactions [44,45]. As a result, they create favorable conditions for microbial growth, leading to an overall increase in enzymatic activity, including CA [46]. The relatively high CA observed in soils under lupine stands, both under oil contamination and in combination with other pollutants, may play an important role in the remediation of hydrocarbon-contaminated soils. This can be attributed to the fact that redox enzymes, such as catalase, are involved in the initial stages of organic compound degradation, including oil hydrocarbons, by using their components as electron donors and facilitating the reduction of reactive enzyme intermediates [47,48]. In addition, oxygen released during H_2_O_2_ decomposition may support the activity of aerobic microbial communities involved in oil degradation.

Invertase facilitates the hydrolysis of sucrose into glucose and fructose, which serve as readily available energy substrates for plants and soil microorganisms [49]. Anthropogenic soil pollution can decrease [50], increase [51], or have no significant effect on invertase activity [52,53]. In the present study, the IA value was stable regardless of the type of pollution. It is likely that differences in invertase sensitivity obtained by different authors are due to both the composition and concentration of pollutants and differences in the physicochemical properties of soils [54,55]. Furthermore, there is evidence that invertase activity is closely related to soil moisture [56]. Soil moisture was maintained at a constant level throughout the experiment. This could also explain the stability of invertase activity across all types of contamination. It is possible that the IA under field conditions will differ slightly from the results obtained in the laboratory experiment.

Urease facilitates the hydrolysis of nitrogen-containing organic matter and plays an important role in nitrogen transformation within the soil. The observed decrease in UA under oat crops with the addition of oil has already been described previously [57]. This reduction is attributed to the alteration in the C:N ratio in the soil upon oil introduction, which reduces nitrogen availability for soil microorganisms and plants. The presence of nickel increased UA in the soil under plant stands (Figure 1c). This metal serves as a cofactor for urease [58], and its addition to both clean and oil-contaminated soil may have stimulated enzyme activity. The enhancement in UA under lupine (*Fabaceae*) stands with oil addition aligns with findings from other researchers. For instance, significantly higher UA was observed in oil-contaminated soil under the legume *Lotus corniculatus* compared to oil-free seeded soil [59].

Phosphatase hydrolyzes organic phosphorus-containing compounds into inorganic phosphorus, which is essential for plants. In this study, as well as in the work referenced in [60], copper inhibited PA, while nickel did not have a significant effect on it. Against the background of oil contamination, both metals suppressed the activity of the enzyme (Figure 1d).

Soil contamination affects the abundance and composition of microbial communities, ultimately leading to changes in SBR [61]. Its enhancement in soil with heavy metals is likely associated with the microorganism’s energy requirements for survival and metabolic reorganization, resulting in more active substrate utilization [62,63]. The increased production of carbon dioxide in oil-contaminated soil with oats may have been linked to the development of hydrocarbon-oxidizing microorganisms and the active degradation of hydrocarbons to end products [64] (Figure 1d).

The use of plants in the present experiment resulted in a relatively high degree of oil biodegradation in the soil (33–46%) (Figure 4). This indicator depends on various factors, including soil biological activity, which in the current experiment largely depended on the type of phytoremediants used. Under combined contamination, the inhibition of oat growth (Figure 2) likely served as the primary reason for the reduced rate of oil degradation in the soil under this plant. Overall, the addition of additional pollutants did not affect the efficiency of oil biodegradation in soil with lupine, and the slight stimulation of this process in the «sulfate + oil» treatment may have been associated with the high catalase activity in this sample, which is known to initiate the oxidation of hydrocarbons [65]. The level of soil toxicity to plants depends on the concentration of contaminants present.

In laboratory conditions, the use of test plant seeds allows for the rapid assessment of acute phytotoxicity in soil. Germination index values below 50% are considered indicative of phytotoxic conditions, 50–80% suggest moderate phytotoxicity, and values above 80% indicate the absence of phytotoxicity [31]. According to this rating, following phytoremediation, soil samples were classified as non-toxic or moderately toxic to radish test plants.

Oil contamination and salinization can be considered as multifactorial stresses that include both toxicological and osmotic components. On one hand, hydrocarbons exert direct phytotoxic effects on plant cells (through damage to photosynthetic pigments, proteins, lipids, chloroplasts, microtubules, cell walls, membranes, and nucleic acids [66,67]). Additionally, soil salinization leads to ion imbalance, resulting in increased intracellular concentrations ions (of carbonate and sulfate anions along with their associated sodium (Na^+^) and magnesium (Mg^2+^)) cations, which ultimately disrupts cellular metabolism [68]. On the other hand, oil pollution impairs soil water retention properties [69]. This effect is compounded by dehydration and reduced turgor pressure in plant cells due to excessive salt concentration [70], further exacerbating the water status of test plant sprouts. Consequently, our selected phytoremediants were unable to fully mitigate soil toxicity within a short timeframe (21 days) under combined contaminated “oil + salinization”. Perhaps, with a longer experiment (in this case, the technical conditions were not available), oats and lupine could have provided greater phytoremediation efficiency. Ultimately, this could have resulted in a lack of soil toxicity for the test plants. However, this hypothesis requires experimental confirmation.

The absence of toxic effects on test plants in soil with nickel ions or with their combination with oil under oat stands may be due to the fact that remediant plants could accumulate nickel ions. Furthermore, the metal’s cations may bind to organic matter in the soil, thereby becoming less available for radish plants.

Combined application of oil and heavy metals resulted in lower toxicity compared to single heavy metal contamination because soil particles could become coated with oil, thus hindering metal ion penetration into the soil solution. Moreover, it has been reported that divalent metal ions can associate with carboxyl groups present in oil [71].

In our view, relatively low levels of soil toxicity are likely attributable to positive phytoremediation effects exerted by the growing oat and lupine plants. These beneficial outcomes may arise from a synergistic interplay of several factors, including root exudate synthesis, the stimulation of rhizosphere microbial growth involved in contaminant degradation, transformation of heavy metals into biologically unavailable forms (such as via chelation processes), and partial uptake of heavy metals by plants themselves [72,73,74,75].

Plant morphological responses enable the early detection of damage caused by pollution and can be used as indicators of the presence of soil contaminants. Overall, shoot growth in both plant species was most strongly suppressed by oil and its combinations with other pollutants. This effect can be attributed to a reduction in the soil’s water-holding capacity due to oil contamination and, consequently, to insufficient water supply to plants [76] (Figure 2a). In addition, all tested contaminants may have exerted direct toxic effects, manifested as inhibited growth and the development of aboveground plant organs.

As for the roots, their growth in the studied plants was most significantly inhibited by copper ions (Figure 2b). In oats, this metal accumulates first in the roots and then in the shoots [40], while lupine has a limited ability to absorb and low translocation of copper from roots to shoots [77]. These plant strategies toward copper are largely determined by the nature and intensity of root exudates. The presence of chelating agents (low-molecular-weight organic acids and flavonoids) in root exudates and the high exudation rate in lupine are mechanisms that prevent copper uptake by roots [78]. When copper levels in the environment are excessive, its ions can penetrate the roots and bind to phenolic compounds in the root apoplast. The synthesis of phytosiderophores and phenolic acids by oat roots also promotes copper binding in the rhizosphere. However, their production may decline over time, triggering other detoxification mechanisms, including copper immobilization in the root symplast [41]. The root is the first line of defense against copper in the soil; therefore, the underground part of the plants is the first to experience the toxic effects of excess copper [79]. Additionally, elevated concentrations of this metal can lead to changes in the balance of other macro- and microelements in plants [80] and thus limit their growth [41].

In both plant species, no symptoms indicative of the phytotoxic effect of nickel were observed. As a rule, this metal has low mobility and phytoavailability in soils [81]. It is possible that part of the added nickel was in a form inaccessible to plants due to the complexation of metal ions with organic matter in the soil.

MDA is the main cytotoxic product of lipid peroxidation and can serve as an indicator of the formation of lipid radicals. Its concentration in tissues reflects the degree of oxidative stress in plants [82].

According to the data, the greatest stress on oats was caused by copper contamination of the soil. Copper, being an oxidatively active metal, is known to directly induce the formation of active oxygen species through the Fenton and Haber–Weiss reactions [83]. A significant increase in the amount of MDA in plants under the influence of excess copper concentrations has already been noted by other authors [84].

In lupine, the highest level of MDA was caused by soil contamination with oil. Oil is known to be a source of multifactorial abiotic stress [85]. The deterioration in soil physicochemical properties due to its presence can cause stress in plants due to a lack of water, oxygen, and nutrients. Furthermore, oil components can have a direct toxic effect on plant cells. All of this can lead to oxidative stress through indirect mechanisms such as interactions with the antioxidant system, disruption of electron transport, and the induction of lipid peroxidation. The results of this study are consistent with previous studies, which also noted that the amount of MDA in leguminous plants significantly increased when the soil was contaminated with hydrocarbons [86,87].

Chlorophyll plays an important role in the processes of photosynthesis and biomass accumulation in plants. It is more sensitive to changes in external conditions than other pigments, so its content in leaves can serve as an objective indicator of stress caused by environmental factors [88].

The decrease in chlorophyll content in the leaves of remediant plants under soil contamination with oil may be associated with the fact that hydrocarbons can lead to chlorophyll degradation [89]. Soil contamination with nickel, both alone and in combination with oil, led to a decrease in chlorophyll quantity in both plant species (Table 2). This could be related to the negative impact of high concentrations of this metal on photosynthetic activity and the amount of photosynthetic pigments in the leaves due to disruptions in membrane permeability, chloroplast ultrastructure, as well as CO_2_ fixation processes and electron transport mechanisms [42,90]. The reduced formation of chlorophyll in the leaves of oats and lupine in the “oil + sulfate anion” variant can also be explained by the fact that the accumulation of salts in plant tissues under soil salinization can lead to stomatal and non-stomatal limitations of photosynthesis. The latter is associated with disruptions in the structure of the photosynthetic apparatus [91] and abnormalities in chlorophyll biosynthesis [92]. In general, the results of this work are consistent with other reports of a decrease in the content of photosynthetic pigments in the leaves of plants grown on soil contaminated with oil [93], nickel [94] and salts [95]. It is known that the addition of carbonates to acidic soils leads to the immobilization of toxic mobile aluminum compounds, which act as typical root toxins. These compounds cause root damage by destroying cell walls and deforming cells, thereby inhibiting root growth [96,97]. An increase in chlorophyll content in plant leaves, accompanied by a decrease in the proportion of mobile aluminum in soil following carbonate application, has been reported in previous studies [98].

One important function of flavonoids is protecting plants against oxidative stress due to their pronounced antioxidant activity [99]. The increase in flavonoid content in oat leaves following copper application, both alone and in combination with oil, is likely due to the formation of excess amounts of ROS and free radicals as a result of the toxic effect of copper. Due to their electron-donating activity, flavonoids act as antioxidants, neutralizing free radicals. Furthermore, flavonoids can serve as heavy metal chelators to prevent the development of oxidative stress [100]. Apparently, the increase in flavonoid content in oat plants in the presence of copper was one of the mechanisms of protection against oxidative stress. Similar results were obtained by other researchers [101], who noted increased flavonoid formation under conditions of excessive heavy metal levels.

The NBI, defined as the ratio of chlorophyll to flavonoid content, is widely regarded as an indicator of plant nitrogen status [102]. The most pronounced decrease in the NBI in oat leaves under soil contamination with copper indicates reduced nitrogen availability for plants in the presence of this metal. In lupine, oil as a single contaminant caused the greatest reduction in the NBI, which is consistent with previous studies [19,103] demonstrating the negative effects of hydrocarbons on nitrogen availability for plants.

Overall, the results of this study indicate the potential use of oat and lupine plants for the remediation of oil-contaminated soils in the presence of additional pollutants. Since laboratory conditions have their limitations, we plan to conduct field trials in the future to confirm the information obtained in this experiment.

## 5. Conclusions

In the present study, the effects of oil; copper and nickel cations; carbonate and sulfate anions; as well as complex contamination (oil in combination with each of the listed pollutants) on soil biological activity and the condition of phytoremediants—oats and lupine—were investigated. None of the studied types of contamination under plant stands exerted a pronounced toxic effect on the soil microbiota. Catalase, urease, and phosphatase proved to be suitable enzymes for monitoring the soil condition under mono- and binary contamination. Soil basal respiration was an effective indicator for assessing the negative effects of heavy metals and oil on soil. In both plant species, root growth was most strongly inhibited by copper ions, whereas shoot growth was suppressed by oil and its combinations with other pollutants. Analysis of the malondialdehyde content in plant leaves revealed that oats experienced the greatest stress in the presence of copper, while lupine was most affected by oil contamination. Despite the negative physiological and biochemical responses of phytoremediants to the studied contaminants, a significant reduction in soil hydrocarbon content was observed: under oat stands, the degree of hydrocarbon degradation reached 33–46%, while under lupine stands, it was 33–38%. Overall, the results indicate that oat and lupine plants can be effectively used for the phytoremediation of soils contaminated with oil and its combinations with heavy metals and salts, as well as for the development of biological remediation technologies for such territories.

## Figures and Tables

**Table 1:** The number of some ecological–trophic groups of microorganisms, CFU/g.

	Oats			Lupine		
Variants	Heterotrophic, ×10^7^	Hydrocarbon- Oxidizing, ×10^6^	Micromycetes, ×10^5^	Heterotrophic, ×10^7^	Hydrocarbon- Oxidizing, ×10^6^	Micromycetes, ×10^5^
Control	0.92 ± 0.05 ^b^	2.02 ± 0.09 ^e^	0.22 ± 0.01 ^b^	2.02 ± 0.08 ^b^	2.06 ± 0.09 ^a^	0.32 ± 0.01 ^c^
Cu^2+^	0.81 ± 0.03 ^a^	0.11± 0.01 ^a^	4.01 ± 0.31 ^d^	1.06 ± 0.05 ^a^	2.01 ± 0.10 ^a^	0.12 ± 0.01 ^a^
Ni^2+^	0.90 ± 0.04 ^b^	0.21 ± 0.01 ^b^	0.12 ± 0.01 ^a^	5.08 ± 0.26 ^e^	2.63 ± 0.14 ^b^	0.15 ± 0.01 ^a^
CO_3_^2−^	0.83 ± 0.03 ^a^	1.15 ± 0.04 ^d^	0.13 ± 0.01 ^a^	3.04 ± 0.13 ^c^	9.22 ± 0.44 ^d^	0.21 ± 0.01 ^b^
SO_4_^2−^	1.04 ± 0.03 ^c^	1.03 ± 0.03 ^c^	0.11 ± 0.01 ^a^	2.12 ± 0.09 ^b^	4.36 ± 0.21 ^c^	0.33 ± 0.02 ^c^
oil	8.92 ± 0.42 ^g^	10.08 ± 0.52 ^f^	1.20 ± 0.03 ^c^	6.06 ± 0.31 ^f^	10.33 ± 0.45 ^e^	0.92 ± 0.07 ^e^
Cu^2+^ + oil	4.85 ± 0.25 ^d^	1.26 ± 0.06 ^d^	3.76 ± 0.19 ^d^	4.29 ± 0.20 ^d^	21.25 ± 0.12 ^g^	0.63 ± 0.02 ^d^
Ni^2+^ + oil	6.07 ± 0.38 ^e^	10.02 ± 0.55 ^f^	1.15 ± 0.06 ^c^	7.11 ± 0.38 ^g^	32.01 ± 0.16 ^h^	1.26 ± 0.07 ^f^
CO_3_^2−^ + oil	9.06 ± 0.50 ^g^	11.08 ± 0.76 ^f^	1.06 ± 0.05 ^c^	7.26 ± 0.35 ^g^	10.34 ± 0.51 ^e^	0.91± 0.05 ^e^
SO_4_^2−^ + oil	7.03 ± 0.35 ^f^	10.03 ± 0.48 ^f^	1.23 ± 0.06 ^c^	4.14 ± 0.19 ^d^	12.18 ± 0.70 ^f^	1.38 ± 0.08 ^f^

Different letters indicate significant differences in the average values for each parameter (*p* ≤ 0.05). Values in each column are compared.

**Table 2:** Pigment content and NBI in plant leaves.

	Oats			Lupine		
Options Experience	Chlorophyll (μg/cm^2^)	Flavonoids (μg/cm^2^)	NBI (a.e.)	Chlorophyll (μg/cm^2^)	Flavonoids (μg/cm^2^)	NBI (a.e.)
Control	20.9 ± 0.3 ^c^	0.78 ± 0.02 ^c^	26.8 ± 0.7 ^c^	49.2 ± 1.2 ^f^	0.351 ± 0.009 ^c^	140.8 ± 3.5 ^de^
Cu^2+^	20.1 ± 0.3 ^b^	0.91 ± 0.02 ^e^	22.1 ± 0.6 ^a^	48.1 ± 1.2 ^f^	0.344 ± 0.009 ^c^	140.4 ± 3.5 ^de^
Ni^2+^	19.3 ± 0.3 ^a^	0.83 ± 0.02 ^d^	23.4 ± 0.6 ^b^	43.6 ± 1.1 ^e^	0.32 ± 0.008 ^b^	136.3 ± 3.4 ^d^
CO_3_^2−^	23.1 ± 0.4 ^d^	0.70 ± 0.01 ^b^	32.9 ± 0.8 ^d^	49.1 ± 1.2 ^f^	0.329 ± 0.008 ^bc^	148.1 ± 3.7 ^e^
SO_4_^2−^	21.1 ± 0.3 ^c^	0.64 ± 0.02 ^a^	32.4 ± 0.8 ^d^	49.1 ± 1.2 ^f^	0.332 ± 0.009 ^bc^	147.2 ± 3.7 ^e^
oil	19.2 ± 0.3 ^a^	0.70 ± 0.02 ^b^	27.7 ± 0.7 ^c^	28.9 ± 0.7 ^a^	0.380 ± 0.009 ^d^	85.1 ± 2.1 ^a^
Cu^2+^ + oil	23.6 ± 0.4 ^d^	0.85 ± 0.03 ^d^	28.0 ± 0.7 ^c^	34.9 ± 0.9 ^c^	0.331 ± 0.008 ^bс^	112.6 ± 2.8 ^c^
Ni^2+^ + oil	19.1 ± 0.3 ^a^	0.82 ± 0.02 ^cd^	23.4 ± 0.6 ^b^	40.1 ± 1.0 ^d^	0.294 ± 0.007 ^a^	138.2 ± 3.5 ^d^
CO_3_^2−^ + oil	21.5 ± 0.3 ^c^	0.70 ± 0.01 ^b^	31.3 ± 0.8 ^d^	39.0 ± 1.0 ^d^	0.341 ± 0.009 ^c^	114.9 ± 2.9 ^c^
SO_4_^2−^ + oil	19.4 ± 0.3 ^a^	0.72 ± 0.02 ^b^	26.8 ± 0.7 ^c^	30.7 ± 0.8 ^b^	0.332 ± 0.008 ^bc^	94.0 ± 2.4 ^b^

Different letters indicate significant differences in the average values for each parameter (*p* < 0.05, *n* = 9). The values in each column are compared.

**Table 3:** Soil phytotoxicity under oat and lupine stands.

Options Experience	Share Sprouted Seeds, %	Elongation Root, %	Index Germination, %	Phytotoxicity
Oats				
Control	98.3 ± 4.7 ^b^	98.0 ± 4.7 ^c^	96.3 ± 4.7 ^d^	Absent
Cu^2+^	90.0 ± 4.4 ^ab^	88.3 ± 4.2 ^b^	79.5 ± 3.8 ^bc^	Moderate
Ni^2+^	93.0 ± 4.5 ^ab^	93.0 ± 4.4 ^bc^	86.5 ± 4.2 ^c^	Absent
CO_3_^2−^	93.0 ± 4.3 ^ab^	91.2 ± 4.5 ^bc^	84.8 ± 4.0 ^c^	Absent
SO_4_^2−^	86.6 ± 4.1 ^a^	72.6 ± 3.5 ^a^	62.9 ± 3.2 ^a^	Moderate
oil	91.6 ± 4.5 ^ab^	94.1 ± 4.6 ^bc^	86.2 ± 4.5 ^c^	Absent
Cu^2+^ + oil	90.0 ± 4.1 ^ab^	91.2 ± 4.2 ^bc^	79.4 ± 3.8 ^bc^	Moderate
Ni^2+^ + oil	90.4 ± 4.4 ^ab^	93.0 ± 4.3 ^bc^	84.1 ± 4.1 ^c^	Absent
CO_3_^2−^ + oil	88.3 ± 4.2 ^a^	86.0 ± 4.2 ^b^	75.9 ± 3.6 ^b^	Moderate
SO_4_^2−^ + oil	91.2 ± 4.5 ^ab^	86.2 ± 4.3 ^b^	78.6 ± 3.7 ^bc^	Moderate
Lupine				
Control	97.2 ± 4.8 ^c^	98.0 ± 4.5 ^c^	95.3 ± 4.8 ^d^	Absent
Cu^2+^	80.3 ± 4.1 ^ab^	82.0 ± 4.0 ^ab^	65.9 ± 3.2 ^a^	Moderate
Ni^2+^	85.1 ± 4.2 ^b^	84.1 ± 4.1 ^ab^	71.6 ± 3.6 ^ab^	Moderate
CO_3_^2−^	93.3 ± 4.6 ^bc^	87.3 ± 4.2 ^b^	81.5 ± 3.9 ^c^	Absent
SO_4_^2−^	93.3 ± 4.5 ^bc^	88.0 ± 4.3 ^b^	82.1 ± 4.0 ^c^	Absent
oil	76.2 ± 3.5 ^a^	96.1 ± 4.7 ^c^	73.2 ± 3.5 ^b^	Moderate
Cu^2+^ + oil	93.3 ± 4.7 ^bc^	88.0 ± 4.1 ^b^	82.1 ± 4.2 ^c^	Absent
Ni^2+^ + oil	94.2 ± 4.5 ^c^	96.4 ± 4.6 ^c^	90.8 ± 4.5 ^d^	Absent
CO_3_^2−^ + oil	90.0 ± 4.5 ^bc^	88.0 ± 4.2 ^b^	79.2 ± 3.8 ^bc^	Moderate
SO_4_^2−^ + oil	94.2 ± 4.6 ^c^	79.6 ± 4.0 ^a^	75.0 ± 3.7 ^bc^	Moderate

In each column, the differences in mean values between different letters are determined (*p* ≤ 0.05).

## Data Availability

The original contributions presented in the study are included in the article, and further inquiries can be directed at the corresponding author.
